# Spatio-temporal ditribution and transmission dynamics of sheep pox and goat pox diseases in South Wollo zone north East Ethiopia

**DOI:** 10.1016/j.heliyon.2024.e27470

**Published:** 2024-03-04

**Authors:** Belege Tadesse, Sileshi Aregahagn, Bethelihem Tegegne Muluneh, Yalelet Worku

**Affiliations:** a Wollo University, School of Veterinary Medicine, P.O. Box. 1145, Dessie, Ethiopia; b Kombolcha College of Agriculture Affliated to Wollo University, Kombolcha, Ethiopia; c Department of Veterinary Laboratory Technology, College of Agriculture, Food and Climate Science, Injibara University, Injibara, Ethiopia

**Keywords:** *Distribution*, *Kutaber*, *GP*, *SP*, *South wollo zone*, *Spatio-temporal*, *Transmission dynamics*

## Abstract

Sheep pox (SP) and goat pox diseases (GP) are highly transmittable, malignant systemic and economically significant caused by the genus *Capripoxvirus*. using The spatio-temporal distribution of SP and GP outbreaks in South Wollo zone from September 2013 to December 2019 was determined retrospectively using SP and GP outbreaks report Kombolcha regional laboratory. A follow up study was also conducted from December 2019 to March 2021 to estimate the transmission parameter of SP and GP outbreaks in South Wollo zone of Kutaber district, Amhara region. Tissue samples from outbreaks in Kundi and Haroye kebele of Kutaber district were taken to confirm the outbreak by conventional polymerase chain reaction (PCR). The transmission parameters were estimated using Generalized linear model (GLM) based on stochastic Susceptible Infected and Recovered (SIR) model. In South Wollo zone, 249 SGP outbreaks were reported from 2013 to 2019. The incidence differed between months, with a highest peak in October and November and a lowest peak in February. The basic reproduction ratios (R0) of the SGP disease outbreaks were 1.84 and 3 for Haroye and Kundi kebele outbreaks, respectively. The disease is distributed throughout the zone and the investigated active outbreaks had moderate transmission between animals. Hence, it needs a great effort which focuses on the application of control measures that reduce the transmission of the disease.

## 1 Introduction

Sheep and goats are source of cash income and foreign currency and play a vital role as sources of main livelihoods for smallholder keepers in Ethiopia [1]. As reported by Mueller et al. [2], in Ethiopia 63%, 25% and 12% of the meat production was gained from cattle, sheep and goat, respectively. The 92% and 90% of the skin and live animal export were gained from sheep and goats, respectively. Tefera [3] also reported that in central highlands of Ethiopia in which mixed crop-livestock production system is experienced, sheep and goats account cash income of 40% and household meat consumption of 19%. However, studies indicate that this contribution to the national economy is below the potential [4]. From the major factors which hinder the production and productivity of small ruminants; SP and GP are grouped from the pinnacle diseases [5].

GP and SP are highly contagious diseases of goats and sheep [6] caused by the genus Capri poxvirus, one of the six genera of poxviruses of vertebrates [7]. SP and GP are characterized by fever, discharge from eye and nose, respiratory distress, abortion, reluctant to move and generalized nodular skin lesions [8]. The disease can cause a morbidity of 75–100% and case fatality can range from 10 to 85% depending on the virulence of the virus [9]. SP and GP disease leads economic losses due to reduction in productivity, death, abortion, skin rejection and baning from international trade [6,7,[14], [15], [16], [17]].

SP and GP spreads through direct contact with infectious animals and indirect contact with contaminated objects like dried scabs and wool [10]. A number of studies showed that the majority of SP and GP outbreaks occur during the winter and spring months [7,11].

SPand GP are extensively distributed in many countries including Asia, North and East Africa [5]. In Ethiopia, the diseases were reported from Amhara, Oromia and Afar regions with different levels of antibodies and outbreaks prevalence. The difference in outbreaks prevalence is mainly due to the movement of sheep and goats.

These diseases are among the most important diseases of sheep and goats in Ethiopia including Amhara National Regional State (ANRS) [[12], [13], [14]], Afar region [19] and Oromia region [21] following Peste des petits ruminants (PPR) and contagious caprine pleuropneumonia (CCPP). Fentie et al. [14] has reported 15.5% overall sero prevalence of SP and GP in Western Amhara region in a mixed population of sheep and goats and 548 outbreaks with 22, 888 cases and 2477 deaths in the whole Amhara region [14]. An other study in Eastern Amhara region reported 663 SP and GP disease outbreaks with 16,825 cases and 1334 deaths [22]. Vaccination is available for the diseases but some districts are irregularly addressed and they fall under an outbreak. The surveillance of SP and GP diseases was not well organized in Ethiopia and assessment of both diseases and their status are practiced when suspected outbreaks are reported to the veterinary authority of the respected district.

To understand the transmission of SP and GP virus and to predict its transmission dynamics, quantification of SP and GP virus transmission parameters is indispensable. Quantification of transmission parameters for SP and GP can be performed either by using field outbreak data or by using data from animal experiments [18]. Quantitative information on the distribution of SP and GP is also essential in order to make sound decisions about its control.

Even if there are some reports on the sero prevalence and risk factors of SP and GP in selected areas of Afar region, Eastern Amhara region and Western Amahara region and molecular characterization of the viruses in central Ethiopia [14,[19], [20], [21], [22]], the transmission parameters are still not estimated in areas with similar agroecology to the current outbreak areas. The spatial and temporal patterns of the SP and GP outbreaks in South Wollo zone are also not well studied in depth. A better perception of its transmission parameters and distribution are very important inputs to select effective and economically feasible disease control measures. Therefore, this study was conducted to determine the spatial and temporal distribution and estimate the per day transmission rate parameter and basic reproduction ratio of SP and GP outbreak in South Wollo zone.

## 2 Materials and methods

### 2.1 Description of the study areas

**Fig. 1:**
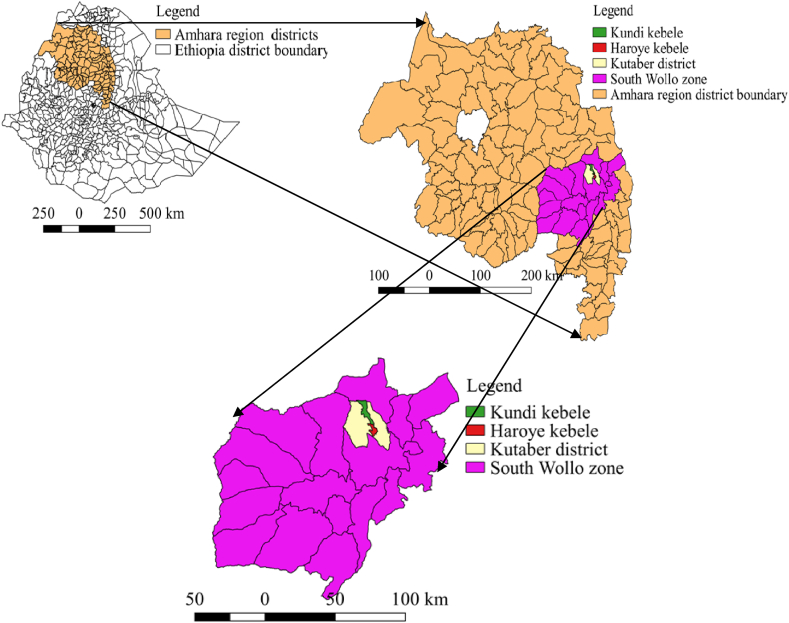
Map of the study area (Produced by using QGIS 2.18.28).

The spatio-temporal study was done in all districts of South Wollo zone, whereas, the transmission dynamics study was conducted in Haroye and Kundi kebeles (the smallest administrative unit of Ethiopia) of Kutaber district of South Wollo zone (Fig. 1). The zone is the set of twenty four administrative districts including administrative towns which is located in the north eastern part of Ethiopia between 9°20′ and 14°20′ North latitude and 36° 20′ and 40° 20′ East longitude and Kutaber district is also located between 11° 19′ 60″ North latitude and 39° 14′ 60″ East longitude. The altitude of this district ranges from 800 m above sea level (m.a.s.l) at the northernmost point to 3200 (m.a.s.l) in the hilly areas [23].

### 2.2 Study design

A retrospective data was used to determine the spatial and temporal distribution of GP and SP outbreaks in South Wollo zone, whereas, field follow up of outbreaks from December 2019 up to March 2021 was used to estimate the transmission rate parameters of the outbreaks.

### 2.3 Study population

A total of 1, 373,184 sheep and 826,468 goats population [24] and 71490 sheep and 49615 goats are reared in South Wollo zone and Kutaber district (KDLRA, 2021), respectivelly. From such numbers of sheep and goats that are reared in kutaber district 3062 sheep and 1935 goats were in Haroye kebele and 3311 goats and 583 sheep were kept in Kundi kebele. In the study area, goats and sheep are the main livelihoods of farmers.

### 2.4 Sample size and sampling techniques

Spatio-temporal study: All SP and GP outbreaks reported from South Wollo to the Kombolcha regional veterinary laboratory from September 2013 to December 2019 were taken to study the spatio-temporal distribution of SP and GP outbreaks in the zone. In South Wollo zone, all the SP and GP outbreaks in the zone were reported to Kombolcha regional veterinary laboratory. Therefore,a total of 249 SP and GP outbreaks reported to Kombolcha regional veterinary laboratory from South Wollo zone from September 2013 to December 2019 were included.

Transmission dynamics study: Haroye and Kundi kebeles in Kutaber district were selected purposively for the study based on the availability of outbreaks during the study period for the transmission dynamics study. Within the selected Kebeles,the whole flocks (sheep and goats of all breeds, sex and age) were included in the study and followed. Accordingly, a total of nine flocks with 181 sheep and goats and eleven flocks with 299 sheep and goats were included from Kundi and Haroye kebele outbreaks, respectively.

### 2.5 Data collection

#### 2.5.1 Temporal and spatial distribution

The data for the determination of the spatial and temporal distribution of outbreaks of SP and GP in South Wollo zone was collected from the Epidemiology unit of Kombolcha regional veterinary laboratory for the period September 2013 to December 2019. The records in the data included information such as location, species affected, index date, number of cases, number of outbreaks, number of deaths, number of vaccine given, and number of animals at risk.

#### 2.5.2 Quantification of transmission parameters

##### 2.5.2.1 Outbreak follow-up and infection dynamics data collection

For the estimation of transmission parameters; data collected by following an outbreak was used because passive surveillance data may lead exaggeration or low estimation of parameters. Affected district and kebeles were identified by continuous and intimated communication to the study district animal health experts and based on outbreak reports. In Kebele's affected by the outbreak, flocks were visited once per week to check whether or not sheep and goats showing symptoms of SGP were present until the end of the outbreak. If a new case was found in a flock, the infection chain within the flock was monitored by visiting the affected flock twice a week until the end of the outbreak and the SP and GP status (susceptible, infected and recovered) of all sheep and goats were recorded. Flock owners were asked to record new cases in each day and report to kebele's animal health experts and the researcher.

At the beginning of the study, all sheep and goats in the affected kebele (i.e. one epidemiological unit) were assumed to be susceptible. In this study, sheep and goats were considered infected when they showed clinical signs of SP and GP during the follow up period of the outbreak. Sheep and goats were registered as a new case on the date they were reported or seen with SGP clinical signs and as infectious on the next day [25,26]. Infected sheep and goats were assumed to stay infectious on average for ten days [26] by taking into account the duration of capripox virus isolation in blood and oro-pharngeal (OP) fluid. Sheep and goats that were treated and isolated from the flock were excluded from the infectious compartment. Tissue samples (nodules from different parts of the body) were collected from clinically infected sheep and goats and the cause of the outbreak was identified using conventional PCR. The conventional PCR was done up on its standard protocol to amplify a small fragment of the 30 KDa RNA polymerase sub unit RP030 gene [27].

##### 2.5.2.2 Quantification of SGP diseases transmission rate parameters

The transmission rate parameter was estimated based on a susceptible-infectious-recovered (SIR) epidemic model in which individuals are either susceptible (S), infectious (I) or recovered and immune (R). During the study, the numbers of infectious, susceptible and recovered sheep and goats observed in each flock was recorded at the start of each observation interval. Transmission of SGPV between sheep and goats was estimated from the relationship between the number of infectious sheep and goats at the start of the time interval and the number of newly infected sheep and goats at the end of the time interval. Every new infection is related to the number of sheep and goats that is infectious at the time of infection. The transmission rate parameter (β) was estimated by a generalized linear model (GLM) [[28], [29], [30]] (based on a stochastic SIR epidemic model [31,32] in which transmission of SGPV between individuals were described by the change in the number of susceptible, infectious and recovered animals. In SIR model, susceptible animal become infected with a rate of:

d*S*/d*t* = - β*I (*t*)*S (*t*)/*N* (*t*);

where β is the transmission rateper day, St, It and Nt are the number of susceptible, infectious and total number of sheep and goats at time t, respectively. Each of the parameters in the above formula is very important to determine the number of sheep and goats that will become infected from the susceptible ones after each visit. The transmission rate (β) between sheep and goats was determined as β = e^b^, in which b is the coefficient of the intercept of the GLM model [32] using complementary log log-LINK function, number of new cases as dependent variable, the natural logarithm of (I*Δ*t*/*N*) as the offset variable and S as binomial total. R0 was determined by multiplying the average infectious period of SP and GP virus infected sheep and goats with β [30,31].

### 2.6 Data analysis

The collected data obtained from laboratory and filed investigation have been edited; filtered, coded using Microsoft Excel spreadsheet 2007 and analyzed by using STATA version 14. The spatial and temporal distribution of SP and GP outbreaks were presented using tables and figures. Statistically significant differences were recorded at p < 0.05 with 95 % confidence interval.

## 3 Results

### 3.1 Temporal distribution of SGP outbreaks in South Wollo zone

A total of 249 laboratory confirmed GP and SP outbreaks were reported from September 2013 to December 2019, to Kombolcha regional veterinary laboratory from South Wollo zone of Eastern Amhara region. High number of SP and GP outbreaks were reported in 2015 (n = 85 outbreaks) followed by 2016 (n = 71) and 2018 (n = 35), while the lowest number of outbreaks were reported in 2014 (n = 6) (Table 1). The outbreaks were higher in the months starting from the end of summer season to dry cold season (spring) compared to other seasons. The highest number of outbreaks were reported in the month of October (n = 40 across all years), which accounted for 16% of all reported outbreaks and the lowest in February (n = 7), accounting for 3% of all reported outbreak. There was no significant difference in the occurrence of SP and GP outbreaks between months (p = 0.25) and years (p = 0.22) (Table 2). In general the number of SP and GP disease outbreaks was above average for the months September to January and below average for February to August. The average occurrences of SP and GP disease in each month was 20.75 (Table 2).

**Table 1:** Annual distribution of SP and GP outbreaks in South Wollo zone.

Year (GC)	No. of SGP outbreaks	P- value	Chi-square
2013	9	0.22	42
2014	6		
2015	85		
2016	71		
2017	18		
2018	35		
2019	25		
Total	249		

Note: GC = Gregorian Calendar.

**Table 2:** Monthly occurrence of SGP disease in South Wollo zone.

Months	No. of outbreaks	P- value	Chi-square
**September**	26	0.25	108
**October**	40		
**November**	36		
**December**	21		
**January**	21		
**February**	7		
**March**	19		
**April**	14		
**May**	16		
**June**	17		
**July**	18		
**August**	14		
**Total**	249		
**Average outbreak per month**	20.75		

A total of 249 SGP outbreaks with 4628 cases and 600 deaths were reported in South Wollo zone from September 2013 to December 2020 (Table 3) with relatively low level of mortality every year. The number of susceptible population, incidence and case fatality of SP and GP varies between each year.

**Table 3:** Annual incidence, mortality and case fatality of SGP outbreaks in South Wollo zone from September 2013–December 2019.

Year	No of Outbreaks	No of death	Susceptible population	No of cases	Incidence of SGP (%)	Mortality (%)	Case fatality (%)
**2013**	9	9	35889	58	0.16	0.03	16
**2014**	6	3	22622	62	0.27	0.01	5
**2015**	85	310	559279	2795	0.50	0.06	11
**2016**	71	177	201434	918	0.46	0.09	19
**2017**	18	20	58828	156	0.27	0.03	13
**2018**	35	64	75250	325	0.43	0.09	20
**2019**	25	17	54713	314	0.59	0.03	5
**Total**	249	600		4628			12.6

### 3.2 Spatial distribution of SGP disease outbreaks in South Wollo

**Fig. 2:**
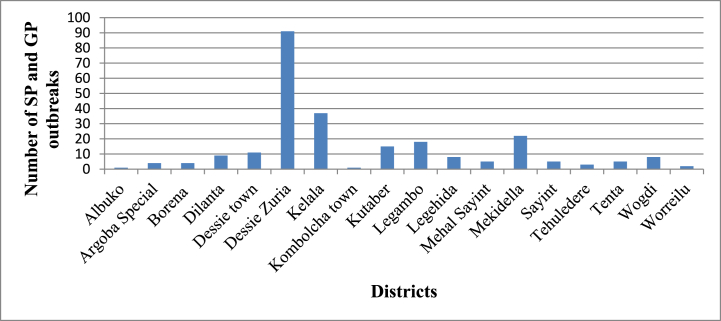
The distribution of SP and GP in South Wollo zone districts from September 2013 to December 2019.

During the period September 2013 to December 2019, SGP outbreaks has been reported from the administrative districts (n = 18) of South Wollo zone. More or less such numbers of administrative districts (n = 18) in the South Wollo zone reported more than one SGP outbreak in this time period. In total 249 SGP outbreaks were reported in South Wollo zone from all districts (Table 1). Most of these outbreaks were from Dessie zuria district (37%, n = 91), Kelala district (15%, n = 37) and Mekdela district (9%, n = 22) (Fig. 2).

### 3.3 Quantification of transmission parameters

The cause of the outbreaks in Kundi and Haroye kebele of Kutaber district of South Wollo zone was confirmed as GP virus. The basic reproductive ratios (R0) for Kundi and Haroye kebeles were 3 and 1.84 respectively. There was a significant difference in the transmission of the disease between individual infected sheep and goats within the outbreak (P = 0.000), whereas, there was no significant different in transmission between the two outbreaks (P = 0.09) (Table 4).

**Table 4:** Transmission rate parameters of SGP outbreak in Kundi and Haroye Kebele.

Kebeles	β(95% CI)	Ro (95% CI)	p-value (b/n animals within outbreaks)	P- value(b/n outbreaks)
**Haroye**	0.184 (0.15–0.19)	1.84(1.21–2.60)	0.000	0.09
**Kundi**	0.3 (0.25-0.35	3 (1.34–4.02)	0.000	

**Fig. 3:**
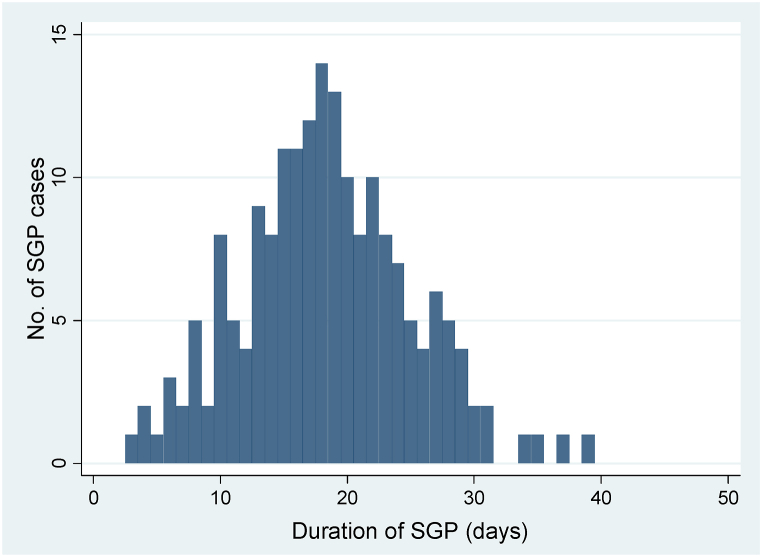
Epidemic curve of SP and GP outbreaks in Kundi kebele, Kutaber, Ethiopia during February–June 2020.

**Fig. 4:**
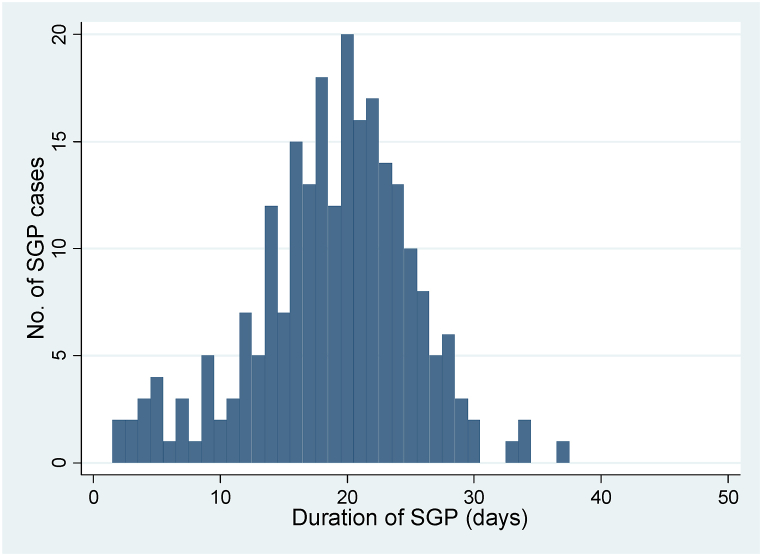
Epidemic curve of SGP outbreaks in Haroye kebele, Kutaber, Ethiopia during February–June 2020.

The maximum or the peak point of the outbreaks were at days 18 **(**Fig. 3) and at day 20 (Fig. 4) for Kundi and Haroye kebele outbreaks respectively from the commencement of the disease.

The vaccination coverage (*Pc*) of SP and GP for Kundi Kebele was *Pc* = (1-(1/Ro); 1-(1/3) = 66.7% and for Haroye Kebele; *Pc* = 1-(1/1.84) = 45.7%

## 4 Discussion

In the current study, SP and GP outbreaks that occurred in South Wollo zone and active outbreaks in kutaber district were investigated through a retrospective and a longitudinal follow-up of cases. There by, the study produced detailed and dependable data on the distribution and transmission dynamics of the disease.

In the current study a total of 249 outbreaks were reported in South Wollo zone from September 2013 to December 2020. Fentie et al. [14] reported 154 and 115 outbreaks in North Shoa and South Wollo in the period from January 2010 to December 2014, which are lower than the current finding. The differences may be due to the difference in the duration of the study, reporting of each and every outbreak of SP and GP by districts, vaccination coverage difference and season of study.

A total of 249 SP and GP outbreaks with 4628 cases and 600 deaths were reported in South Wollo zone from September 2013 to December 2020. The numbers of SP and GP outbreaks reported in this study are less than 548 outbreaks with higher number of cases (22, 888) and deaths (2477) in the whole Amhara region [14] and 663 SP and GP outbreaks with 16,825 cases and 1334 deaths in Eastern Amhara region as reported by Aregahagn et al. [22]. The variations in the number of outbreaks, cases and deaths are due to the previous study was covering the whole Amhara region and Eastern Amhara region part but the current study covers only one zone (South Wollo) of the region, there also be a difference in the control of the disease.

According to this study, there were a difference in occurrence of SP and GP outbreaks between administrative districts and seasons (months) even though it was not statistically significant. This agrees with the study conducted by AU-IBAR [33], in the greater horn of Africa, Kenya; who reported that seasonality: weather related seasonality and livestock mobility cause stress and can compromise immune response, livestock mobility favors contact between infected and susceptible herds, the presence of naïve populations within an infected region is a major predisposing factor in epidemics, malnutrition, parasitism, bacterial infections, close contact, unregulated trade and porous borders are favoring conditions for emergence of SP and GP outbreaks. As studied by Zangana and Abdullah [34], poor conditioned animals, overcrowding, poor feeding and mismanagement and rough uses of vaccination appeared to be the main reason of distribution and susceptibility to infection with the pox virus. According to Masoud et al. [9], seasonality of the diseases have an impact on the survival of the virusfor longer periods, by association with lambing season and transportation of sheep and goats for marketing.

In few numbers of districts of South Wollo zone there was small number of outbreaks of sheep and goat pox disease. However, this may be due to underreporting of outbreaks by their administrative kebeles or districts, which results in the under estimation of the outbreaks. Bayissa and Bereda [35] reported that communication and transport problems affected the disease reporting in Ethiopia, especially in remote health posts.

Fentie et al. [14] suggested, the failure of vaccination would likely to be caused by poor vaccine handling where the availability of electricity is limited for keeping cold chain. In rare case, since the vaccine is provided by the government itself, some unethical and negligence professionals will not used the vaccine whenever the vaccinating areas are very far from the clinic. This results in the occurrence and spreading of the disease.

In the current study, the incidence of SP and GP outbreaks were in all months of the year; which agree with the report of Atalla and Alzuheir [36] which has done in Palestine. In this study the highest outbreak has been recorded during dry cold season (October, n = 40) followed by November, n = 36) while the lowest was at February. Atalla and Alzuheir [36], reported highest number of outbreaks in January and fewest outbreaks and lowest incidence and mortality in August and September, respectively. This might be due to seasonal and geographical differences between the two study areas. Bhanuprakash et al. [11,37] also reported that SP and GP outbreak occurred in all months of the year, but the highest number of outbreaks and the greatest mortality occurred in March; the fewest outbreaks and the lowest incidence, mortality occurred in August in India. The variation might be due to difference in the management andcontrol methods applied in the countries.

The average numbers of secondary infection (R0) of SP and GP outbreaks were 1.84 and three (3) for Haroye and Kundi kebele, respectively. This describes that a sheep and goats virus will continue its propagation among susceptible members of the sheep and goats, if no environmental changes or external influences intervene. This finding is higher as compared to the report of Tadesse et al. [38] in Chifra districts of Afar region, who reported a R0 of 1.41. The values of R0 indicate that a single SP and GP virus infected animal can transmit for 1.84 sheep and goats for Haroye kebele and three sheep and goats for Kundi kebele's respectively. The current finding is also higher than the R0 of 1.21 (95% CI: 1.01–1.46) reported by Molla et al. [26] from LSD outbreaks in Ethiopia.The variation in the transmission might be due to the difference in the structure of the population, species, geographical area and season of the outbreaks.

In the current study the peak numbers of sheep and goats that were getting infected in each outbreaks were at day 18 and day 20 for Kundi and Haroye kebele SP and GP outbreak respectively, which agrees with the report of Tadesse et al. [22] in Chifra districts of Afar region.

## 5 Conclusion and recommendations

Sheep pox and GP disease is widely spread and endemic in South Wollo zone. It has been occurred in this zone except a few administrative districts in the time period between September 2013 to December 2019 GC. Outbreaks varies in months and more outbreaks were occurred in October. GP virus was identified as the cause of the current outbreaks in Kutaber district. In both outbreaks a moderate transmission rate were estimated. Therefore, to reduce its distribution and transmission it requires future effort which hub on the application of control measures including vaccination and control of animal movement and early respose for SP and GP outbreaks.

## Ethics approval

Not applicable.

## Consent for publication

Not applicable.

## Funding

This research was funded by Wollo University.

## Declaration

We declare that the information presented here in our work is original, and all sources of materials used for the research work have been duly acknowledged.

## Data availability statement

The data used to support the findings of this study are included within the article.

## CRediT authorship contribution statement

**Belege Tadesse:** Writing – review & editing, Supervision, Conceptualization. **Sileshi Aregahagn:** Writing – original draft, Resources, Investigation. **Bethelihem Tegegne Muluneh:** Investigation. **Yalelet Worku:** Writing – original draft.

## Declaration of competing interest

The authors declare that they have no known competing financial interests or personal relationships that could have appeared to influence the work reported in this paper.

## Abbreviations

GC: Gregorian Calendar
GLM: Generalized linear model
GP: goat Pox
PCR: Polymerase Chain Reaction
SP: Sheep Pox
